# Fibroblast Growth Factor-2 Primes Human Mesenchymal Stem Cells for Enhanced Chondrogenesis

**DOI:** 10.1371/journal.pone.0022887

**Published:** 2011-07-27

**Authors:** Andrew M. Handorf, Wan-Ju Li

**Affiliations:** Department of Orthopedics and Rehabilitation and Department of Biomedical Engineering, University of Wisconsin-Madison, Madison, Wisconsin, United States of America; University of Southern California, United States of America

## Abstract

Human mesenchymal stem cells (hMSCs) are multipotent cells capable of differentiating into a variety of mature cell types, including osteoblasts, adipocytes and chondrocytes. It has previously been shown that, when expanded in medium supplemented with fibroblast growth factor-2 (FGF-2), hMSCs show enhanced chondrogenesis (CG). Previous work concluded that the enhancement of CG could be attributed to the selection of a cell subpopulation with inherent chondrogenic potential. In this study, we show that FGF-2 pretreatment actually primed hMSCs to undergo enhanced CG by increasing basal Sox9 protein levels. Our results show that Sox9 protein levels were elevated within 30 minutes of exposure to FGF-2 and progressively increased with longer exposures. Further, we show using flow cytometry that FGF-2 increased Sox9 protein levels per cell in proliferating and non-proliferating hMSCs, strongly suggesting that FGF-2 primes hMSCs for subsequent CG by regulating Sox9. Indeed, when hMSCs were exposed to FGF-2 for 2 hours and subsequently differentiated into the chondrogenic lineage using pellet culture, phosphorylated-Sox9 (pSox9) protein levels became elevated and ultimately resulted in an enhancement of CG. However, small interfering RNA (siRNA)-mediated knockdown of Sox9 during hMSC expansion was unable to negate the prochondrogenic effects of FGF-2, suggesting that the FGF-2-mediated enhancement of hMSC CG is only partly regulated through Sox9. Our findings provide new insights into the mechanism by which FGF-2 regulates predifferentiation hMSCs to undergo enhanced CG.

## Introduction

Due to the limited capacity of articular cartilage for self repair and lack of effective treatments for cartilage injuries and degenerative joint diseases like osteoarthritis [1], tissue engineering has gained considerable attention as a promising approach to cartilage repair. Tissue engineering approaches aim to recapitulate normal developmental and tissue healing processes to drive tissue regeneration. For tissue engineering strategies to be successful, an appropriate cell source must be instructed to proliferate and then differentiate by specific bioactive cues, such as growth factors and extracellular matrix molecules. Human mesenchymal stem cells (hMSCs) provide an attractive cell source for cartilage tissue engineering applications, as they are readily expandable [2] and capable of differentiating into chondrocytes [3]. However, hMSCs represent a heterogeneous population of cells with varying chondrogenic potential [4], [5], thus limiting their use in cartilage tissue engineering applications. In addition, hMSCs fail to produce the quality or quantity of cartilage matrix compared to articular chondrocytes under identical conditions [6]. Thus, improvements to our current expansion conditions will be necessary to purify those hMSCs with greatest chondrogenic potential and/or enhance their ability to undergo chondrogenesis (CG) if cartilage tissue engineering using hMSCs is to be of clinical relevance.

Fibroblast growth factor-2 (FGF-2) is one of over 20 members of the large FGF family of heparin-binding polypeptide growth factors. FGF-2 acts as a potent mitogen for several cell types of mesenchymal origin [7], [8], [9]. In addition, FGF-2 has been implicated in the regulation of several key signaling cascades involved in the development and maintenance of cartilage, including the earliest stages of limb development [10], [11]. *In vitro*, it has previously been shown that, when expanded in culture medium supplemented with FGF-2, hMSCs show enhanced chondrogenic potential [8]. It is unclear, however, how FGF-2 enhances the CG of hMSCs. Two potential mechanisms may be operating individually or in tandem: FGF-2 treatment may select a subpopulation of hMSCs with inherent chondrogenic potential to proliferate (referred to as selection) [12]; or FGF-2 may change the molecular machinery of the entire population or a subset of the population in some way to more efficiently respond to chondrogenic cues, thus enhancing subsequent differentiation (referred to as priming) [5].

Previous work has suggested that, in response to FGF-2, hMSC CG is enhanced through the selection of an immature stem cell population with inherent multipotentiality, as FGF-2 had equally potent effects in enhancing hMSC osteogenesis [13] and adipogenesis [14]. Additionally, it was shown that expansion of hMSCs in FGF-2-supplemented medium led to an initial increase in average telomere length without detectable telomerase activity, suggesting that FGF-2 selects for the survival and expansion of a subset of cells enriched in multipotent precursors [12]. While these studies present a convincing case for a selection-based mechanism driving the observed enhancement of CG, other studies have suggested greater complexity beyond simply the selection of a multipotential stem cell population. For instance, Adesida *et al.* reported that the redifferentiation of dedifferentiated chondrocytes, a presumably homogeneous cell population, is greatly enhanced by expansion in FGF-2, as well. In fact, FGF-2 enhanced the re-expression of *collagen type II (Col II)* 200-fold upon subsequent differentiation in pellet culture [15].

Sox9, an SRY-related transcription factor harboring the conserved high-mobility group DNA-binding domain, is required for successive steps of cartilage formation during development. At early stages, Sox9 functions to initiate chondrogenic lineage specification in a population of mesenchymal precursor cells that condense to form precartilaginous buds [16]. Inactivation of *Sox9* in precursor cells prior to condensation leads to the complete absence of cartilage formation [17]. Later during development, Sox9 binds directly to regulatory elements of cartilage-specific genes, such as *Col II* and *aggrecan (AGN)*, to drive chondrogenic differentiation [18], [19]. Sox9 also activates the expression of *L-Sox5* and *Sox6* [17], transcriptional co-activators that are likewise required for articular cartilage formation [20].

In the current study, we investigated further the mechanism underlying the observed FGF-2-mediated enhancement of CG. We begin by offering new insights into the nature by which the FGF-2-treated hMSC population is modulated after long-term exposure and extend upon these findings to show that 2 hours of exposure to FGF-2 was sufficient to enhance hMSC CG, thus suggesting a priming-based mechanism for the observed enhancement of CG. FGF-2 increased basal Sox9 protein levels in hMSCs within 30 minutes, and continued exposure to FGF-2 led to progressively increased Sox9 protein levels. Importantly, FGF-2 increased Sox9 protein levels in both proliferating and non-proliferating hMSCs, further suggesting that FGF-2 primes hMSCs for subsequent CG by regulating Sox9. However, small interfering RNA (siRNA)-mediated knockdown of Sox9 was unable to negate the enhancement of CG induced by FGF-2, suggesting that FGF-2 enhances hMSC CG only partly through Sox9. Nonetheless, FGF-2 was a potent inducer of Sox9 expression and there is a strong correlation between Sox9 levels prior to CG and the effectiveness of differentiation.

## Results

### FGF-2 enhances hMSC CG and modulates lineage-specific transcription factor expression

**Figure 1:**
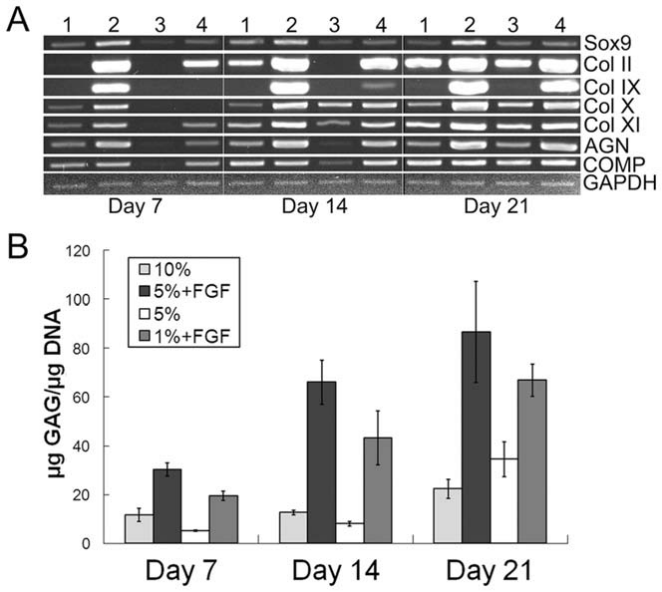
FGF-2 enhances hMSC CG. Human MSCs were expanded in varying concentrations of FBS with or without FGF-2 for 2 passages prior to CG and analyzed for cartilaginous properties upon differentiation in pellet culture. **(A)** End-point RT-PCR analysis showed that cartilage-specific gene expression was upregulated in the 2 FGF-2-expanded conditions (Lanes 2 and 4) at each time point compared to those without FGF-2 pretreatment (Lanes 1 and 3). Lane 1: 10% FBS; Lane 2: 5% FBS+FGF-2; Lane 3: 5% FBS; Lane 4: 1% FBS+FGF-2. **(B)** Significantly greater GAG accumulation was observed in the 2 FGF-2-expanded conditions at each time point compared to those without FGF-2 pretreatment. GAG content was normalized to DNA content. Values are mean ± SD. All conditions were significant with each other except the 2 FGF-2-expanded conditions at Day 21. p<0.05, n = 3.

Expanding hMSCs in medium supplemented with FGF-2 enhanced subsequent differentiation into the chondrogenic lineage compared to non-FGF-2-supplemented controls, as evidenced by the upregulation of cartilage-specific gene expression (Fig. 1A) and GAG accumulation (Fig. 1B). Specifically, both hMSCs expanded for 2 passages in 5%- (Fig. 1A; Lane 2) and 1% FBS+FGF-2 (Fig. 1A; Lane 4) showed upregulated cartilage-specific gene expression compared to cells expanded in 10% FBS (Fig. 1A; Lane 1), the current gold standard for hMSC expansion, upon chondrogenic induction at each time point. Importantly, *Col II* showed relatively high gene expression as early as Day 7 in both FGF-2-expanded cultures, whereas *Col II* expression was not detected until Day 14 in 10% FBS-expanded cells and Day 21 in 5% FBS-expanded cells (Fig. 1A; Lane 3). Similarly, *collagen type IX* (*Col IX*) was expressed at detectable levels earlier in both FGF-2-expanded cultures, appearing at Days 7 and 14 in 5%- and 1% FBS+FGF-2-expanded cells, respectively. Strikingly, *Col IX* was not expressed at detectable levels even at Day 21 of differentiation in either of the non-FGF-2-expanded hMSC populations. Similar trends were observed when quantifying GAG accumulation normalized to DNA content within chondrogenic pellets (Fig. 1B). After 21 days of differentiation, both FGF-2-expanded hMSC populations produced significantly more GAG (86.6 and 66.9 µg GAG/µg DNA) than the non-FGF-2-expanded control cells (22.4 and 34.5 µg GAG/µg DNA) (p<0.05). These data clearly demonstrate that FGF-2 has potent effects in enhancing the chondrogenic potential of predifferentiation hMSCs.

**Figure 2:**
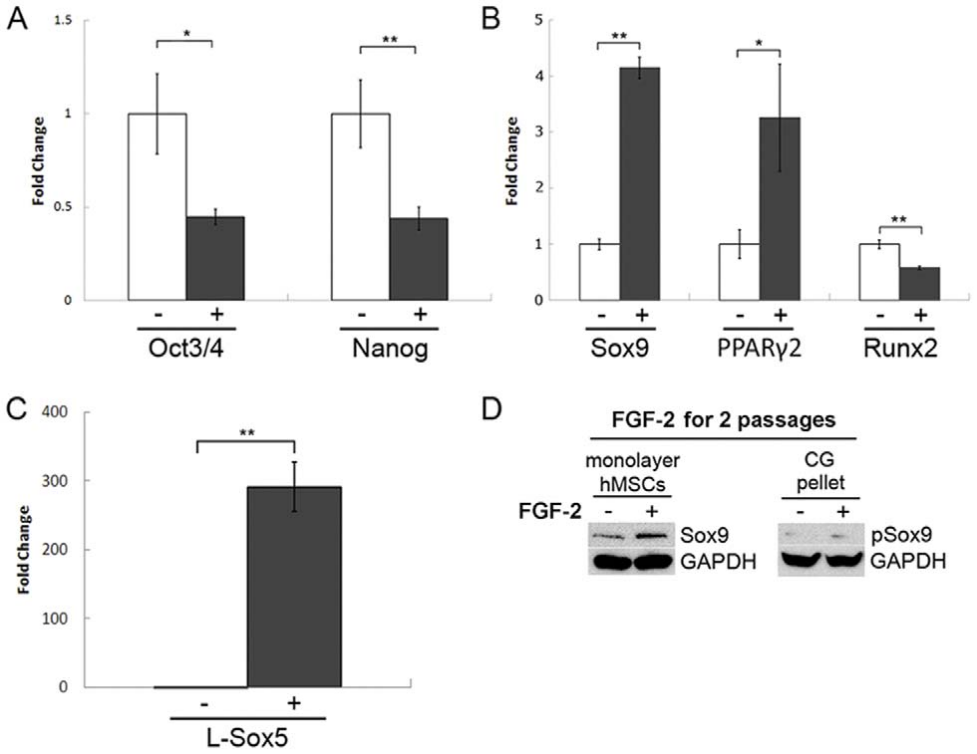
FGF-2 reduces potency and modulates lineage-specific transcription factor expression of hMSCs. Human MSCs were expanded for 2 passages in 5% FBS with (+) or without (−) FGF-2 supplementation and assayed for gene and protein expression prior to pellet culture. **(A, B, C)** Real-time RT-PCR analysis demonstrated that FGF-2-expanded hMSCs significantly downregulated the expression of embryonic stem cell markers (A), modulated the expression of key lineage-specific transcription factors (B), and upregulated the expression of *L-Sox5* (C). All values were normalized to GAPDH expression and compared to the non-FGF-2-expanded control. Values are represented as mean ± SD. *p<0.05, **p<0.005, n = 3. **(D)** Western blot analysis showed that FGF-2-expanded hMSCs exhibited higher Sox9 protein levels than non-FGF-2-expanded hMSCs prior to CG, and after pellet formation, pSox9 protein levels were present at higher levels. GAPDH was used as a loading control.

To investigate the nature by which FGF-2 alters the phenotype of hMSCs prior to chondrogenic induction, hMSCs were expanded in medium containing 5% FBS with or without FGF-2 for 2 passages and analyzed for gene and protein expression using real-time RT-PCR and Western blotting, respectively. Human MSCs maintained in FGF-2-supplemented medium showed greatly modulated gene expression prior to chondrogenic differentiation (Fig. 2A–C). Specifically, FGF-2 significantly downregulated *Oct3/4* and *Nanog*, stem cell pluripotency markers (Fig. 2A), upregulated *Sox9* and *PPARγ2*, transcription factors that regulate chondrogenic- and adipogenic-specific gene expression (Fig. 2B), and downregulated *Runx2*, a transcription factor that regulates osteogenic-specific gene expression (Fig. 2B). In addition, FGF-2 led to the dramatic upregulation of *L-Sox5* expression, which showed a 292-fold increase in mRNA transcript levels (Fig. 2C). Importantly, the observed upregulation in *Sox9* gene expression with FGF-2 supplementation translated to an increase in Sox9 protein levels after expansion for 2 passages (Fig. 2D). Upon chondrogenic induction in pellet culture, phosphorylated-Sox9 (pSox9) protein levels became elevated 12 hours after pellet formation in hMSCs expanded in FGF-2 (Fig. 2D). Together, these data suggest that FGF-2 drives hMSCs to leave the multipotent state and move toward the chondrogenic lineage. Further, the observed phenotypic changes persist even after the onset of differentiation, suggesting that the increase in Sox9 protein levels may act to enhance subsequent CG.

### FGF-2 enhances hMSC CG through both selection- and priming-based mechanisms

**Figure 3:**
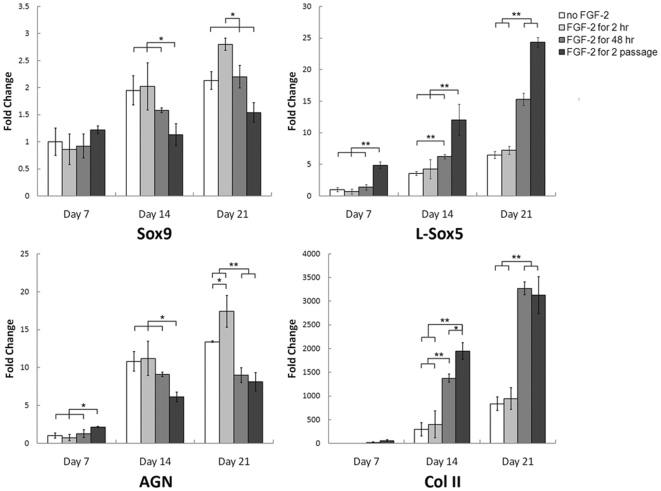
FGF-2 enhances hMSC CG through both selection- and priming-based mechanisms. Human MSCs were expanded for 2 passages in medium supplemented with FGF-2 for varying durations of time and assayed for cartilage-specific gene expression in pellet culture up to 21 days using real-time RT-PCR. Longer durations of FGF-2 exposure during hMSC expansion resulted in the significant upregulation of *L-Sox5* and *Col II* gene expression, while exposure to FGF-2 for just 2 hours resulted in the significant upregulation of *Sox9* and *AGN* by Day 21. All values were normalized to GAPDH expression and compared to the non-FGF-2-expanded control at Day 7. Values are represented as mean ± SD. *p<0.05, **p<0.005, n = 3.

To investigate the mechanism by which FGF-2 enhances CG, hMSCs were expanded for 2 passages in medium supplemented with FGF-2 for varying durations of time. Given that a selection-based mechanism relies on cell proliferation to manifest its effects, if it were indeed operating, longer exposures to FGF-2 would allow for the progressive enhancement of CG due to the continual selection of a subpopulation of hMSCs with inherent chondrogenic potential. Since hMSCs divide with an average doubling time of 12–24 hours in response to a mitogenic stimulus [21], hMSCs were exposed to FGF-2 for 48 hours and 2 passages (referred to as selection-based conditions). On the other hand, if a priming-based mechanism were operating, exposing hMSCs to FGF-2 for short durations of time, where cell proliferation specifically in response to FGF-2 can be assumed negligible, would result in an enhancement of CG. In this study, hMSCs were exposed to FGF-2 for 2 hours prior to the onset of chondrogenic differentiation (referred to as priming-based condition). As a control, hMSCs were maintained in basal medium containing 5% FBS without FGF-2 supplementation. After expansion, hMSCs were differentiated into the chondrogenic lineage for 21 days and assessed for gene expression of *Sox9*, *L-Sox5*, *AGN* and *Col II* (Fig. 3). For each of the 4 genes, the priming-based condition showed reduced mRNA transcript levels at Day 7, while the selection-based conditions showed a trend of enhanced CG. At Days 14 and 21, the gene expression results showed two patterns. For *Sox9* and *AGN*, the priming-based condition showed a gradual upregulation in gene expression, culminating in significantly higher mRNA transcript levels than the control and selection-based conditions by Day 21, whereas for *L-Sox5* and *Col II*, the selection-based conditions showed significantly higher mRNA transcript levels than the priming-based condition and control hMSCs at Days 14 and 21. While insignificant, the priming-based condition also showed consistently higher *L-Sox5* and *Col II* transcript levels than the control at Days 14 and 21. Together, these data suggest that both selection- and priming-based mechanisms are operating to enhance hMSC CG in response to FGF-2. However, it does not eliminate the possibility that hMSCs are progressively primed after longer exposures to FGF-2 or show how FGF-2 regulates hMSCs for enhanced CG.

### FGF-2 primes hMSCs for CG by increasing basal Sox9 protein levels

**Figure 4:**
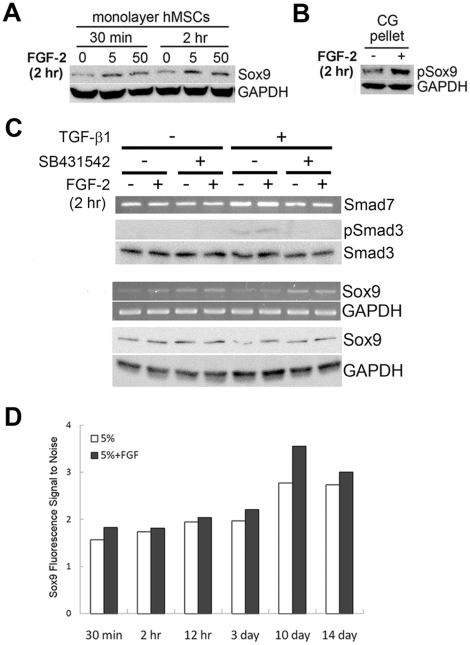
FGF-2 primes hMSCs for CG by increasing basal Sox9 protein levels. **(A)** Human MSCs were exposed to FGF-2 for 30 minutes or 2 hours and analyzed for Sox9 protein levels prior to pellet culture by Western blot analysis. Exposure to 5 and 50 ng/mL FGF-2 led to increased Sox9 protein levels in a dose-dependent manner. GAPDH was used as a loading control. **(B)** Human MSCs were exposed to FGF-2 for 2 hours and chondrogenically-induced in pellet culture. FGF-2-expanded hMSCs (+) showed elevated pSox9 protein levels by Western blot analysis. GAPDH was used as a loading control. **(C)** Human MSCs were treated with or without SB431542 for 15 minutes, exposed to combinations of 5 ng/mL FGF-2, 10 ng/mL TGF-β1 and 10 µM SB431542 for 2 hours, and analyzed for Sox9 mRNA and protein levels by end-point PCR and Western blot analysis, respectively. Exposure to FGF-2 led to increased Sox9 mRNA and protein levels, while TGF-β1 reduced Sox9 expression. Smad7 gene and pSmad3 protein levels were analyzed to confirm successful knockdown of TGF-β signaling. GAPDH was used as a loading control. **(D)** Human MSCs were expanded in FGF-2 for varying durations of time and analyzed for Sox9 protein using flow cytometry. Sox9 protein levels per cell were elevated at all time points after FGF-2 exposure compared to the control and gradually increased with longer exposures. Values are represented as fluorescence signal to noise ratios using geometric means.

We next investigated how the phenotype of hMSCs was modulated by FGF-2 after short exposures prior to chondrogenic induction. To do so, we maintained hMSCs in basal medium containing 5% FBS for 2 passages, washed them twice with DPBS to remove residual growth factors and administered FGF-2. The control group again received medium containing 5% FBS without FGF-2, while the 2 treated groups received medium containing 5% FBS with either 5 or 50 ng/mL FGF-2. Remarkably, Sox9 protein levels were elevated in hMSCs prior to differentiation after just 30 minutes exposure to FGF-2 and persisted for at least 2 hours (Fig. 4A), with hMSCs receiving medium supplemented with 5 ng/mL FGF-2 showing a larger increase in Sox9 protein levels than those receiving 50 ng/mL FGF-2. Importantly, hMSCs receiving 5 ng/mL FGF-2 just 2 hours prior to chondrogenic induction in pellet culture showed substantially higher pSox9 protein levels 12 hours after chondrogenic induction compared to non-FGF-2-exposed control hMSCs (Fig. 4B), suggesting that hMSCs are indeed primed by FGF-2 prior to chondrogenic induction, and these phenotypic changes are pivotal for enhancing CG.

Since Sox9 expression has been shown to be upregulated by TGF-β signaling in a variety of contexts [22], [23], [24], we explored whether FGF-2 increases Sox9 expression through crosstalk with the TGF-β signaling pathway. To do so, hMSCs were treated with or without 10 µM SB431542, a specific inhibitor of TGF-β receptor 1 kinase activity, for 15 minutes. Human MSCs were subsequently cultured in medium containing 5% FBS with or without FGF-2, TGF-β1, and SB431542 for 2 hours. In the presence of TGF-β1, gene expression of Smad7, a direct target of TGF-β signaling, was upregulated, and Smad3 phosphorylation was induced without affecting total Smad3 protein levels (Fig. 4C). However, when cotreated with SB431542, Smad7 mRNA and Smad3 phosphorylation levels were reduced to basal levels, demonstrating successful knockdown of TGF-β signaling. End-point PCR and Western blot analysis showed that inhibition of TGF-β signaling increased Sox9 mRNA and protein levels, while treatment with TGF-β1 caused a substantial decrease in Sox9 expression. Importantly, treatment with FGF-2 was able to restore Sox9 protein levels to that in hMSCs treated with SB431542 (Fig. 4C), suggesting that FGF-2 does not increase Sox9 levels through crosstalk with the TGF-β signaling pathway.

To gain greater mechanistic insight into how FGF-2 regulates hMSCs after short- and long-term exposure, we analyzed Sox9 protein levels on a per cell basis using flow cytometry after varying durations of exposure to FGF-2. Flow cytometry analysis for Sox9 confirmed that expansion of hMSCs in medium supplemented with FGF-2 increased Sox9 protein levels at all time points compared to non-FGF-2-exposed control hMSCs (Fig. 4D). Notably, Sox9 protein levels per cell gradually increased with longer exposures to FGF-2. Further, the flow cytometry data showed only 1 signal peak in Sox9 fluorescence at all time points, suggesting that all (or a vast majority of) hMSCs are responding to FGF-2 with elevated Sox9 protein levels, whereas multiple diverging signal peaks would be suggestive of a differential response between subpopulations of hMSCs and thus a selection-based mechanism (Fig. S1). Together, these data suggest that hMSCs are continually primed with longer exposures to FGF-2.

### FGF-2 elevates Sox9 protein levels in both proliferating and non-proliferating hMSCs

**Figure 5:**
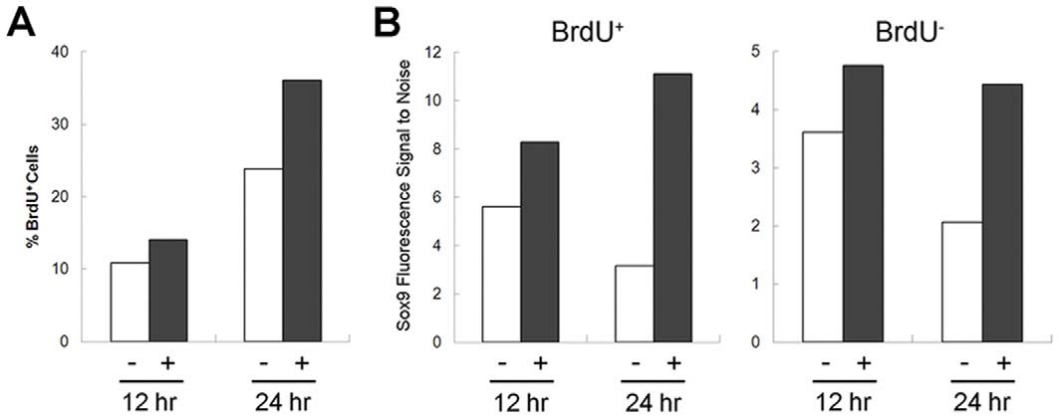
FGF-2 elevates Sox9 protein levels in both proliferating and non-proliferating hMSCs. Human MSCs were exposed to FGF-2 for 12 and 24 hours in the presence of BrdU, double-stained for Sox9 and BrdU, and analyzed using flow cytometry. **(A)** FGF-2 exposed hMSCs showed a higher percentage of BrdU^+^ cells than non-FGF-2-exposed hMSCs after 12 and 24 hours. **(B)** Sox9 protein levels were elevated in both BrdU^+^ and BrdU^−^ FGF-2-exposed hMSCs after 12 and 24 hours, with BrdU^+^ hMSCs showing more marked differences compared to non-FGF-2-exposed control hMSCs. Values are represented as fluorescence signal to noise ratios using geometric means.

To further discriminate between priming- and selection-based mechanisms, we analyzed Sox9 protein levels in proliferating and non-proliferating hMSCs upon FGF-2 induction. Elevated Sox9 protein levels in non-proliferating hMSCs would strongly support the notion that a priming-based mechanism is indeed operating to enhance CG, as selection relies on cell proliferation to manifest its effects. To do so, we incubated FGF-2- and non-FGF-2-exposed hMSCs in 10 µM BrdU, double-stained them for Sox9 and BrdU, and analyzed them using flow cytometry. Given that hMSCs tend to show enhanced CG after both short and long durations of exposure to FGF-2, we hypothesized that both proliferating and non-proliferating hMSCs respond to FGF-2 by increasing basal Sox9 protein levels, thus being primed for subsequent CG. After 12 and 24 hours, FGF-2-exposed hMSCs showed a higher percentage of BrdU^+^ hMSCs, indicating a greater proliferative response (Fig. 5A). FGF-2-exposed BrdU^+^ hMSCs showed marginally elevated Sox9 protein levels after 12 hours (∼1.5-fold increase) and substantially higher Sox9 levels after 24 hours (∼3.5-fold increase) (Fig. 5B) compared to non-FGF-2-exposed BrdU^+^ hMSCs. Although the differences in Sox9 protein were not as significant, FGF-2-exposed BrdU^−^ hMSCs also showed higher Sox9 levels after 12 and 24 hours (∼1.3- and 2.1-fold increase, respectively) (Fig. 5B). Taken together, these data further suggest that a priming-based mechanism is acting to enhance hMSC CG in response to FGF-2, with proliferating hMSCs elevating Sox9 protein levels more dramatically.

### FGF-2 enhances hMSC CG partially through a Sox9-mediated mechanism

**Figure 6:**
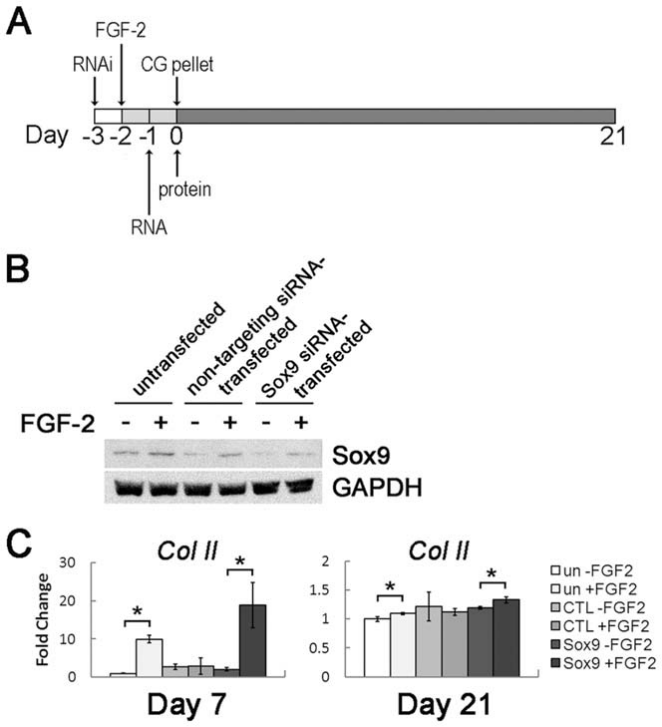
FGF-2 enhances hMSC CG partially through a Sox9-mediated mechanism. **(A)** RNAi experiments were performed to knockdown Sox9 expression. Human MSCs were transfected with siRNA targeted to Sox9 or non-targeting siRNA. 24 hours after transfection, hMSCs received FGF-2, and 72 hours after transfection, hMSCs were differentiated into the chondrogenic lineage in pellet culture. Sox9 knockdown was verified 48 and 72 hours after transfection by gene and protein analysis, respectively. **(B)** Western blot analysis showed that Sox9 levels were lower in both FGF-2- (+) and non-FGF-2-treated (−) hMSCs transfected with Sox9 siRNA compared to untransfected and non-targeting siRNA-transfected hMSCs. With each treatment, FGF-2 increased Sox9 levels. (**C)** After transfected hMSCs were differentiated into the chondrogenic lineage, real-time RT-PCR analysis showed that knockdown of Sox9 expression did not result in a concomitant decrease in *Col II* gene expression 7 and 21 days after pellet formation. However, FGF-2 pretreatment led to a significant increase in *Col II* gene expression in untransfected and Sox9 siRNA-transfected hMSCs.

Finally, to determine whether FGF-2 acts directly through Sox9 to enhance hMSC CG, we used RNA interference (RNAi) to knockdown Sox9. Human MSCs were electroporated with siRNA targeted to Sox9, or with non-targeting siRNA as a control, and then treated with or without FGF-2 24 hours after transfection (Fig. 6A). 72 hours after transfection, hMSCs were induced into the chondrogenic lineage for 21 days using pellet culture. Following transfection, hMSCs displayed a more spindle-shaped morphology than untransfected cells (Fig. S2) and continued to proliferate (data not shown). 48 hours after transfection, *Sox9* gene expression was markedly reduced compared to the negative control, and this correlated to a loss of *Col II* expression in undifferentiated hMSCs (Fig. S3). At the time of chondrogenic induction, Sox9 protein levels were likewise markedly reduced in Sox9 siRNA-transfected hMSCs (Fig. 6B). Notably, Sox9 siRNA-transfected hMSCs showed elevated Sox9 protein levels when treated with FGF-2, exemplifying the potent ability of FGF-2 to induce Sox9 expression. Despite successfully knocking down Sox9 in both FGF-2- and non-FGF-2-treated hMSCs, a concomitant decrease in *Col II* expression was not observed upon chondrogenic induction (Fig. 6C). Interestingly, FGF-2-pretreated hMSCs still showed a significant upregulation of *Col II* gene expression during differentiation. Taken together, these data suggest that while FGF-2 consistently leads to elevated Sox9 levels and improved differentiation, hMSC CG is only partially enhanced by regulating Sox9 levels.

## Discussion

Human MSC-based therapies offer attractive alternatives to current treatments for cartilage injuries and other debilitating conditions, such as osteoarthritis, due to the extensive proliferative capacity of hMSCs and their ability to differentiate into chondrocytes. However, several challenges could potentially limit the use of hMSCs to regenerate cartilage. For one, hMSCs represent a heterogeneous population of cells of varying plasticity [4], [5], thus necessitating the purification of specific subpopulations with enhanced chondrogenic potential [25]. In addition, hMSCs lose their differentiation potential upon continued cell passaging due to the onset of senescence, and even at early passages, these cells fail to produce the quality and quantity of cartilage matrix required for tissue engineering applications [6]. To overcome these limitations, it would be ideal to expand hMSCs in a well-defined medium containing soluble factors that both select the correct subpopulation and enhance subsequent differentiation.

In this study, we extended upon previous findings to show that in addition to selecting a subpopulation of cells with inherent chondrogenic potential, FGF-2 primed hMSCs to undergo enhanced CG by increasing basal Sox9 protein levels. In fact, FGF-2 was remarkably potent at elevating Sox9 levels. Within 30 minutes of exposure to FGF-2, Sox9 levels became elevated (Fig. 4A), and with continued exposure to FGF-2, Sox9 levels progressively increased per cell (Fig. 4C). The continual increase in Sox9 levels could potentially indicate that proliferating hMSCs alone respond to FGF-2 by increasing basal Sox9 levels, but remarkably, non-proliferating hMSCs showed a marked increase in Sox9 levels, as well (Fig. 5B). Together, these data clearly demonstrate that FGF-2 increases basal Sox9 protein levels in hMSCs. Similarly, Murakami *et al.* found that FGF-2 enhanced Sox9 gene and protein expression, as well as the activity of a Sox9-dependent enhancer, in mouse primary chondrocytes and C3H10T1/2 cells. Further, they showed that the FGF-2-induced increase in Sox9 expression could be blocked by inhibiting MEK activity, suggesting that the effects are regulated through the MAPK pathway. While their study suggested that the increase in Sox9 was mediated through the MAPK pathway in mouse cells, Sox9 is likely not a direct downstream target gene of FGF signaling in hMSCs, as *Sox9* gene expression was not upregulated in response to FGF-2 when *de novo* protein synthesis was blocked using cycloheximide (data not shown).

Watanabe et al. showed that there exists a crosstalk between Smad and MAPK pathways to drive chondrocyte-specific gene expression in murine ATDC5 cells [26], raising the possibility that FGF-2 elevates basal Sox9 protein levels through crosstalk with the TGF-β signaling pathway. Additionally, TGF-β ligands have been shown to be potent inducers of Sox9 expression in a variety of cellular contexts [22], [23], [24]. However, our data showed that FGF-2 induced Sox9 expression independent of TGF-β signaling. In fact, TGF-β1 markedly reduced Sox9 expression, thus demonstrating an inhibitory role for TGF-β signaling in hMSCs cultured in monolayer. While our work shows an important role for FGF-2 in inducing Sox9 expression, further work is needed to fully elucidate the underlying signaling mechanism.

Importantly, elevated Sox9 protein levels prior to differentiation resulted in a concomitant increase in pSox9 protein levels upon chondrogenic induction (Fig. 2D and 4B), and these increases correlated strongly with enhanced CG. Sox9, a transcription factor harboring the conserved high-mobility group DNA-binding domain, activates chondrocyte-specific marker genes and is thus critical for differentiation into the chondrogenic lineage [18]. Indeed, in the absence of Sox9, there is a complete blockage of chondrocyte differentiation *in vivo* during development [27]. *In vitro*, Sox9 has also been shown to enhance chondrogenic differentiation and redifferentiation. For instance, Tew *et al.* transduced normal and osteoarthritic chondrocytes with *Sox9* and measured their ability to reacquire a chondrocytic phenotype after sequential passage [28]. *Sox9* transduction caused a 10-fold increase in *Col II* expression in osteoarthritic chondrocytes compared to *GFP*-transduced controls in monolayer culture, an increase that was amplified another 10-fold upon redifferentiation in pellet culture. Similarly, Kupcsik *et al.* transduced hMSCs with *Sox9* and observed an increase in cartilage-specific gene expression upon culture in hydrogel scaffolds without any requirement for exogenous growth factor supplementation [29]. Sox9 clearly has an essential role in CG, and here, we identify a growth factor capable of elevating endogenous Sox9 levels in a physiologically relevant manner prior to differentiation.

Given the indispensable role of Sox9 in chondrocyte differentiation, it is surprising that knocking down Sox9 expression did not have an adverse effect on hMSC CG after FGF-2 stimulation. This may suggest that the increase in Sox9 levels is secondary to another more direct cellular response, such as cytoskeletal organization. Previous reports have shown that FGF-2 induces rapid reorganization of the actin cytoskeleton [30], [31], and in agreement with these findings, our preliminary data show that FGF-2 significantly upregulates the expression of *Rac1* while significantly downregulating *RhoA* expression (data not shown). Furthermore, the organization of the actin cytoskeleton has been implicated as having a central role in chondrocyte differentiation [32], [33]. For instance, disrupting the actin cytoskeleton of hMSCs with the actin depolymerizing drug cytochalasin D markedly enhances differentiation into the chondrogenic lineage [34], and more recent work has suggested that actin organization regulates the transcriptional activity of Sox9 through protein kinase A (PKA) [35]. Taken together, the prochondrogenic effects of FGF-2 may be regulated only secondarily through Sox9, with more direct regulation occurring via an actin cytoskeleton-based signaling mechanism. Given that FGF-2 was able to upregulate Sox9 expression even in Sox9 siRNA-transfected hMSCs (Fig. 6B and S3) and the transfections were designed to only transiently knockdown Sox9, it is also possible that the Sox9 knockdown was lost upon FGF-2 treatment and subsequent chondrogenic induction, as TGF-β signaling, cell-cell interactions and 3D environments have all been shown to modulate Sox9 expression and activity [23], [36], [37]. Nonetheless, there appears to be a strong correlation between Sox9 protein levels in hMSCs prior to CG and their ability to differentiate into the chondrogenic lineage.

Many research groups have previously shown that expansion in medium supplemented with FGF-2 enhances hMSC CG [8], [13], [38]. Mastrogiacomo *et al.* first tested the effect of several growth factors individually or in combination with FGF-2 on hMSC CG and osteogenesis and found that FGF-2 had a dominant effect in enhancing subsequent differentiation. In fact, differentiation into both lineages was evident only in those cultures supplemented with FGF-2, suggesting that FGF-2 maintained hMSCs in an immature state allowing their *in vitro* expansion and maintenance of differentiation potential. Similarly, Tsutsumi *et al.* found that FGF-2 markedly extended the life span of hMSCs, thus allowing them to retain their ability to differentiate into chondrocytes, osteoblasts and adipocytes throughout many mitotic divisions. Furthermore, Bianchi *et al.* showed that FGF-2-supplemented hMSC primary cultures displayed an early increase in telomere length followed by a steady shortening, whereas non-FGF-2-supplemented hMSC cultures showed only a steady decline in telomere length without an initial increase. Since telomerase activity was not detectable in their hMSC cultures, the group concluded that FGF-2 selects for the survival and expansion of a particular subset of cells enriched in multipotent precursors. Together, these 3 studies strongly support the notion that FGF-2 selects for a subpopulation of hMSCs with enhanced multipotency. In contrast, our data suggest that FGF-2 actually decreases the potency of hMSCs, given that hMSCs treated with FGF-2 for 2 passages showed a significant downregulation of *Oct3/4* and *Nanog* expression (Fig. 2A). In addition, our data suggest that FGF-2 primes hMSCs for lineage-specific differentiation, as hMSCs maintained in FGF-2-supplemented medium for 2 passages showed a significant upregulation of *Sox9* and *L-Sox5* expression (Fig. 2B and C). Interestingly, FGF-2 progressively increased basal Sox9 protein levels per cell with longer durations of exposure (Fig. 4C). Likewise, FGF-2-treated BrdU^+^ hMSCs showed an increase in Sox9 protein levels per cell from 12 to 24 hours. Taken together, these results suggest that a priming-based mechanism may be acting to enhance hMSC CG instead of a selection-based mechanism, or the priming- and selection-based mechanisms are one and the same. For instance, it is possible that FGF-2 selects a more youthful cell population to proliferate while concomitantly priming them for differentiation into specific lineages. The resulting FGF-2-treated cell population would be younger, as evidenced by longer telomeres and extended lifespan, but with reduced potency, reflecting a more restricted gene expression profile.

In accordance with the study reported by Im *et al.* [38], we have shown that culture medium supplemented with FGF-2 greatly enhances the chondrogenic potential of hMSCs even under reduced serum conditions. Remarkably, expansion in basal medium containing 1% FBS with 5 ng/mL FGF-2 was able to improve the chondrogenic capacity of hMSCs when compared to standard basal medium containing 10% FBS. Figure 1 clearly demonstrates the importance of supplementing medium with FGF-2 when expanding hMSCs for subsequent chondrogenic differentiation. In addition to enhancing cartilage formation, FGF-2 has also been shown by several groups to accelerate the proliferation rate of hMSCs [8], [39]. Together, FGF-2 appears to be a crucial growth factor for the expansion of hMSCs, yielding a large quantity of quality cells for cartilage tissue engineering applications.

### Conclusion

Our results show that FGF-2 treatment prior to CG is critical to enhance the differentiation of hMSCs into chondrocytes. FGF-2 was found to be a potent inducer of Sox9 expression, and there is a strong correlation between Sox9 levels prior to CG and the effectiveness of differentiation. Thus, FGF-2 primes hMSCs for enhanced CG, at least in part, by increasing basal Sox9 protein levels.

## Materials and Methods

### Ethics Statement

Written consent was obtained from patients by the University of Wisconsin Hospital to obtain bone marrow-derived hMSCs before proceeding with total hip replacement surgeries.

### Isolation and culture of bone marrow-derived hMSCs

With approval from the Institutional Review Board of the University of Washington and University of Wisconsin-Madison, bone marrow-derived hMSCs were isolated from femoral heads of patients undergoing total hip replacement surgery. Whole bone marrow was curetted from the interior of the femoral neck and head, washed in Dulbecco's Modified Eagle's Medium (DMEM) (Gibco, Grand Island, NY) and separated from trabecular bone fragments and other tissues using an 18-gauge needle attached to a 25-mL syringe. The bone marrow cells were centrifuged at 1,200 rpm for 5 minutes to separate them from residual adipose tissue and resuspended in Hank's Balanced Salt Solution (HBSS) (HyClone, Logan, UT). Mononucleated cells were isolated via Ficoll density centrifugation at 600 *g* for 30 minutes and washed in HBSS. Cells were then resuspended in DMEM with 1 g/L glucose (DMEM-LG) supplemented with 10% fetal bovine serum (FBS) (Invitrogen, Carlsbad, CA) from selected lots and 1% antibiotics (10,000 I.U/mL penicillin, 10,000 µg/mL streptomycin, 25 µg/mL amphotericin B) (Mediatech, Manassas, VA) and plated in 75 cm^2^ cell culture flasks (Corning, Corning, NY). Cells were maintained at 37°C in a humidified environment with 5% CO_2_. The following day, culture medium was changed to remove non-adherent cells. Subsequent medium changes were made every 3 days. Upon 80% confluence, cells were passaged using 0.05% trypsin/EDTA (Mediatech) and re-plated at a cell seeding density of 1,000 cells/cm^2^. All cells to be used in studies were frozen in culture medium containing 10% dimethyl sulfoxide (DMSO) (Sigma, St. Louis, MO). Cells were thawed and expanded for 2 passages prior to all experiments. During this expansion phase, cells were generally maintained in DMEM-LG containing 5% FBS, with or without 5 ng/mL FGF-2 (R&D Systems, Minneapolis, MN), as this concentration is optimal for hMSC proliferation and subsequent differentiation [40]. Upon 80% confluence, hMSCs were either analyzed for their predifferentiation properties or harvested for pellet culture.

### Chondrogenic differentiation of hMSCs in pellet culture

Upon 80% confluence, hMSCs were trypsinized, counted, and resuspended in serum-free chondrogenic medium composed of DMEM with 4.5 g/L glucose (DMEM-HG) (Gibco) supplemented with 1% antibiotics, 1% ITS (BD Biosciences, Franklin Lakes, NJ), 0.9% sodium pyruvate (Sigma), 50 µg/mL ascorbic acid, 40 µg/mL L-proline, 10^−7^ M dexamethasone, and 10 ng/mL TGF-β1 (R&D Systems). Aliquots containing 2.5×10^5^ cells were distributed in 96-well round-bottomed tube plates and centrifuged at 600 *g* for 5 minutes. Pellets were cultured in an incubator kept at 37°C in a humidified atmosphere with 5% CO_2_. Medium was changed every 3 days. On Days 7, 14 and 21, cell pellets were harvested and analyzed for cartilaginous properties.

### RNA isolation and RT-PCR analysis

For end-point PCR analysis, total RNA was isolated from chondrogenic pellets with 1 mL TRIzol (Invitrogen) according to the manufacturer's protocol. Briefly, after addition of 200 µL chloroform to homogenized samples, RNA was precipitated with 500 µL isopropanol and washed 3 times in 80% molecular biology grade ethanol. RNA pellets were dissolved in 20 µL DEPC-treated water. For real-time PCR analysis, total RNA was isolated from monolayer hMSCs and chondrogenic pellets using RNeasy Kit (Qiagen, Valencia, CA) according to the manufacturer's protocol. In each case, RNA yields were determined based on A_260_ using NanoDrop 1000 Spectrophotometer (Thermo Scientific; Waltham, MA). RNA samples with 260/280 measurements below 1.80 were further purified using Qiagen RNeasy Kit. First strand cDNA was reverse transcribed from 170 ng total RNA for end-point PCR, and 840 and 160 ng total RNA for monolayer hMSCs and chondrogenic pellets, respectively, for real-time PCR using SuperScript III First-Strand Synthesis System (Invitrogen).

For end-point PCR, cDNA was amplified using Eppendorf Thermal Cycler and Platinum Taq DNA polymerase (Invitrogen) with the oligonucleotide primers found in Table S1. The following cycle numbers were used to amplify gene-specific PCR products: cartilage oligomeric matrix protein (COMP) and collagen type XI, 23; AGN, collagen type X and Sox9, 28; Col II, 30; and col IX, 32. All primer annealing temperatures were optimized empirically, and products for optimized PCR runs were visualized by agarose gel electrophoresis to confirm one product of correct size. Glyceraldehyde-3-phosphate dehydrogenase (GAPDH) expression was used as an internal control to normalize the amount of cDNA synthesized per sample. Twenty-five cycles were used to amplify GAPDH. The PCR products were resolved by agarose gel electrophoresis and visualized by ethidium bromide staining on Kodak Image Station 4000R Pro.

For real-time PCR, cDNA was amplified using BioRad C1000 Thermal Cycler and iQ SYBR Green Supermix (BioRad, Hercules, CA) with the oligonucleotide primers found in Table S1. GAPDH gene expression was used as an internal control to normalize the amount of cDNA synthesized per sample, and all values were compared to non-FGF-2-expanded control hMSCs using the 2^−ΔΔCt^ method. All primer annealing temperatures were optimized so that amplification efficiencies fell within 95 and 105%, and single products were confirmed after 40 amplification cycles at the optimized temperature via agarose gel electrophoresis.

### Protein isolation and Western blot analysis

Cell lysates were prepared from trypsinized monolayer hMSCs or chondrogenic pellets using RIPA solubilization buffer (50 mM Tris-HCl (pH 7.5), 1% Nonidet P-40, 0.25% Na-deoxycholate, 150 mM NaCl, 1 mM EDTA, and complete protease inhibitor cocktail (Roche, Madison, WI)). Chondrogenic pellets needed to be physically dissociated with a pestle upon addition of solubilization buffer for more complete protein extraction. After centrifugation at 14,000 rpm for 10 min at 4°C, the supernatant was collected and protein concentrations were determined using BCA Protein Assay Kit (Pierce, Rockford, IL) against a standard curve of bovine serum albumin. Protein samples were subjected to sodium dodecyl sulfate polyacrylamide gel electrophoresis (SDS-PAGE) and subsequently transferred onto PVDF membranes for Western blotting. Membranes were incubated with the following primary antibodies overnight at 4°C: rabbit polyclonal antibody to Sox9 (Abcam; Cambridge, U.K.), rabbit polyclonal to Sox9 (phospho S181) (Abcam), rabbit monoclonal to Smad3 (Cell Signaling; Danvers, MA) and rabbit monoclonal to phospho-Smad3 (Ser423/425) (Cell Signaling). GAPDH was used as an internal loading control and detected with a rabbit monoclonal antibody (Cell Signaling; Danvers, MA). The secondary HRP-conjugated antibody was goat-anti-rabbit (Cell Signaling). The membrane was developed using SuperSignal West Pico Chemiluminescent Substrate (Pierce) on Kodak Image Station 4000R Pro.

### Glycosaminoglycan quantification

Pellets were digested by papain for 24 hours at 60°C after 7, 14 and 21 days of chondrogenic differentiation. Total sulfated glycosaminoglycan (GAG) was then quantified using Blyscan Assay Kit (Biocolor, Westbury, NY) based on 1,9-dimethylmethylene blue binding against a standard curve of chondroitin-6-sulfate according to the manufacturer's protocol. Total GAG values were then normalized to total DNA content using Quant-iT PicoGreen dsDNA Assay Kit (Invitrogen) in triplicate.

### Flow cytometry staining and analysis

Monolayer hMSCs were trypsinized, pelleted and gently resuspended in ice-cold FACS Buffer (0.1% bovine serum albumin/0.1% sodium azide in 1x DPBS). Single cell suspensions were fixed with 2% paraformaldehyde (methanol-free; Polysciences, Warrington, PA) for 10 minutes at room temperature (RT) in the dark and permeabilized in FACS Buffer with 0.1% saponin (Sigma) for 15 minutes at RT. Fixed and permeabilized samples were incubated with the following antibodies for 1 hour at RT: rabbit polyclonal antibody to Sox9 (Abcam) and rabbit IgG isotype control (Abcam). Samples were then incubated with PE-conjugated donkey polyclonal to rabbit IgG secondary antibody (Abcam) for 30 minutes at RT. Samples were washed 3 times and resuspended in FACS Buffer. For BrdU-incubated cells, hMSCs were fixed using FITC BrdU Flow Kit (BD Pharmingen) following the manufacturer's protocol. Data was acquired using Benton Dickinson FACSCalibur cytometer (488 and 633 nm lasers) and analyzed by dividing the fluorescence intensity geometric means by the geometric mean of relevant isotype controls to reduce background fluorescence. For BrdU-incubated cells, gates were used prior to analysis to differentiate between BrdU^+^ and BrdU^−^ cells.

### RNA interference

ON-TARGETplus SMARTpool siRNA containing a mixture of 4 SMART selection-designed siRNAs targeting Sox9 was purchased from Dharmacon (Lafayette, CO). 200,000 hMSCs were electroporated per 10 µL reaction with Sox9 siRNA (100 nM) using the Neon Transfection System (Invitrogen). Transfections with ON-TARGETplus Non-targeting pool (Dharmacon) were performed in parallel as a negative control. Optimal electroporation conditions (1350 V, 20 ms, and 1 pulse) were determined using siGLO Cyclophilin B control siRNA (Dharmacon). Effective Sox9 knockdown was validated by assaying Sox9 gene and protein expression 48 and 72 hours after transfection, respectively.

### Statistical analysis

All quantifiable studies were reported as means ± standard deviations. Statistical analyses were performed using unpaired Student's t-test. P-values ≤0.05 were considered significant.

## Supporting Information

Figure S1 **FGF-2 primes hMSCs for CG by increasing basal Sox9 protein levels.** Human MSCs were maintained in FGF-2 for varying durations of time and analyzed for Sox9 protein using flow cytometry. Flow cytometry histograms showed a larger shift in fluorescence intensity for hMSCs exposed to FGF-2 (right panel) compared to non-FGF-2-exposed control hMSCs (left panel) at each time point. The shift in fluorescence intensity gradually increased with culture time, and there was only a single peak (shaded gray) in fluorescence intensity at each time point. Fluorescent peaks are plotted against the isotype control peak (unshaded).
(TIF)

Figure S2 **FGF-2 enhances hMSC CG partially through a Sox9-mediated mechanism.** Transfected hMSCs were more spindle-shaped than untransfected hMSCs 24 hours after transfection. Scale bar represents 25 µm.
(TIF)

Figure S3 **FGF-2 enhances hMSC CG partially through a Sox9-mediated mechanism.** Sox9 siRNA-transfected hMSCs (dark gray) showed reduced *Sox9* and *Col II* gene expression 48 hours after transfection compared to untransfected (white) and non-targeting siRNA-transfected hMSCs (gray) using real-time RT-PCR analysis.
(TIF)

Table S1 List of end-point and real-time RT-PCR primers.
(TIF)
