# Supplementary figures and images for: Fibroblast Growth Factor-2 Primes Human Mesenchymal Stem Cells for Enhanced Chondrogenesis
Source: PLoS One. 2011 Jul 27;6(7):e22887. doi: 10.1371/journal.pone.0022887 (PMC3144950; doi:10.1371/journal.pone.0022887)

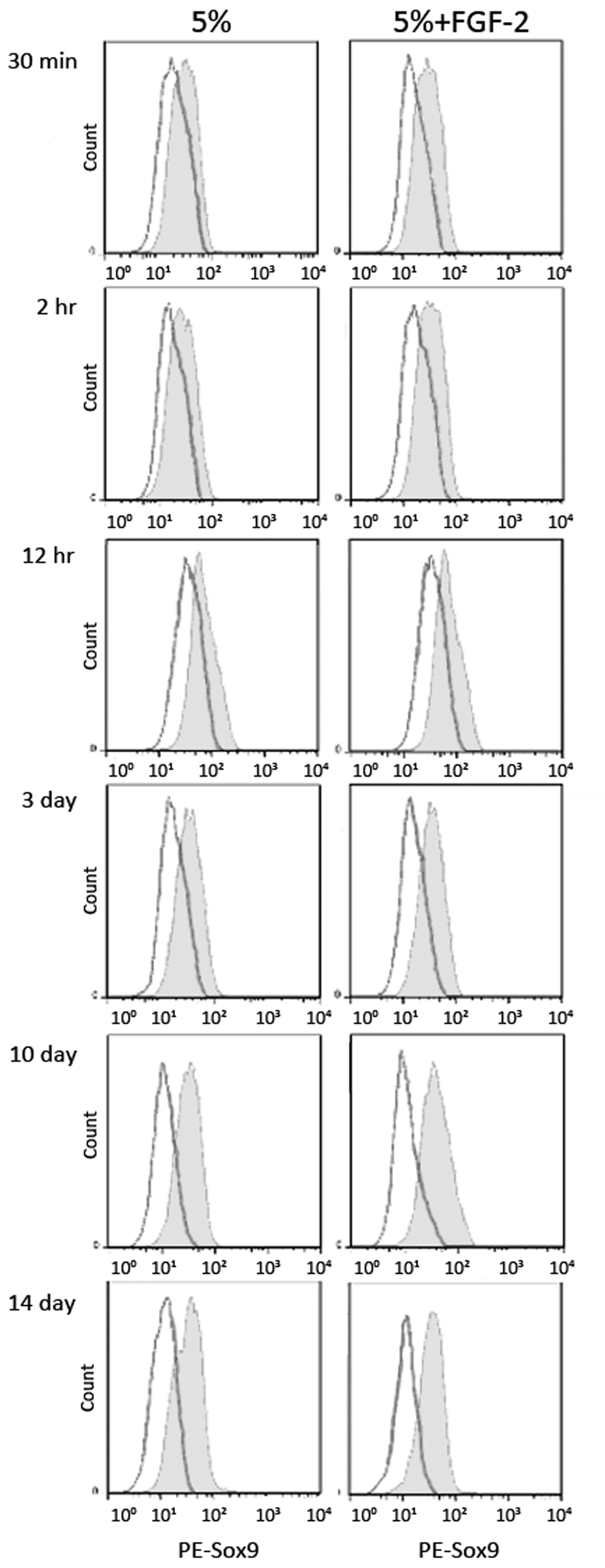

Supplement: Figure S1 — FGF-2 primes hMSCs for CG by increasing basal Sox9 protein levels. Human MSCs were maintained in FGF-2 for varying durations of time and analyzed for Sox9 protein using flow cytometry. Flow cytometry histograms showed a larger shift in fluorescence intensity for hMSCs exposed to FGF-2 (right panel) compared to non-FGF-2-exposed control hMSCs (left panel) at each time point. The shift in fluorescence intensity gradually increased with culture time, and there was only a single peak (shaded gray) in fluorescence intensity at each time point. Fluorescent peaks are plotted against the isotype control peak (unshaded). (TIF) [file pone.0022887.s001.tif]

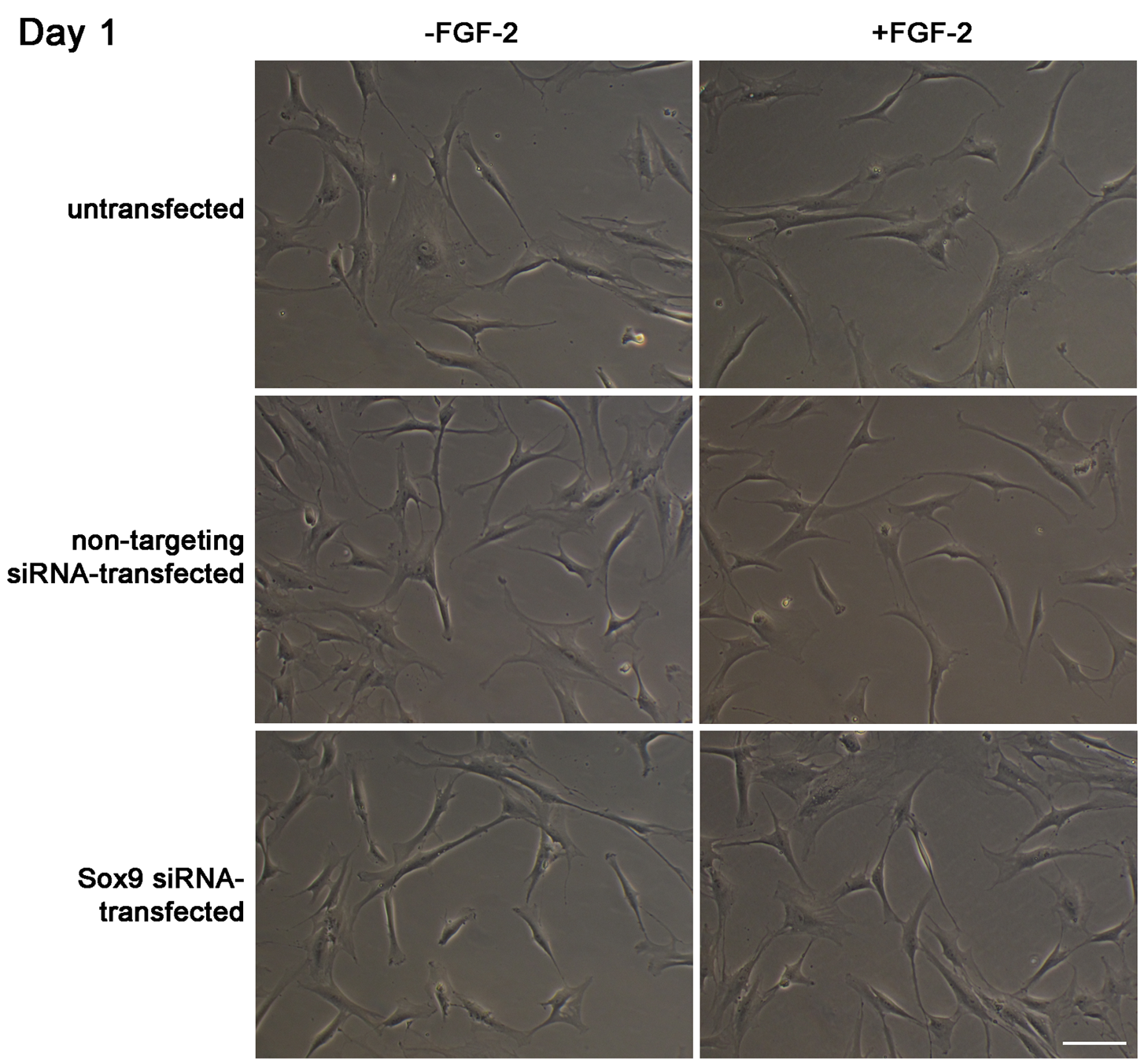

Supplement: Figure S2 — FGF-2 enhances hMSC CG partially through a Sox9-mediated mechanism. Transfected hMSCs were more spindle-shaped than untransfected hMSCs 24 hours after transfection. Scale bar represents 25 µm. (TIF) [file pone.0022887.s002.tif]

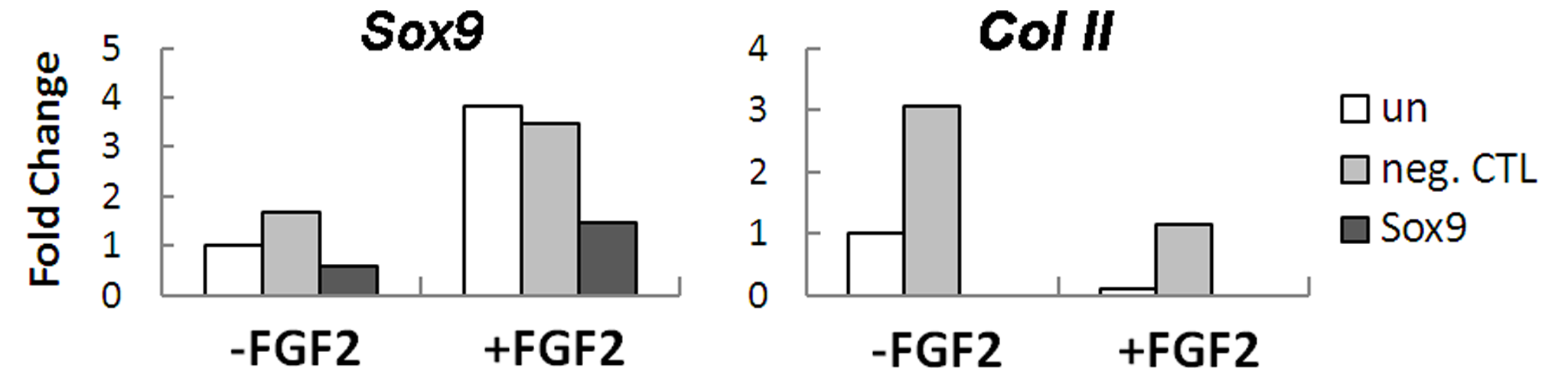

Supplement: Figure S3 — FGF-2 enhances hMSC CG partially through a Sox9-mediated mechanism. Sox9 siRNA-transfected hMSCs (dark gray) showed reduced Sox9 and Col II gene expression 48 hours after transfection compared to untransfected (white) and non-targeting siRNA-transfected hMSCs (gray) using real-time RT-PCR analysis. (TIF) [file pone.0022887.s003.tif]

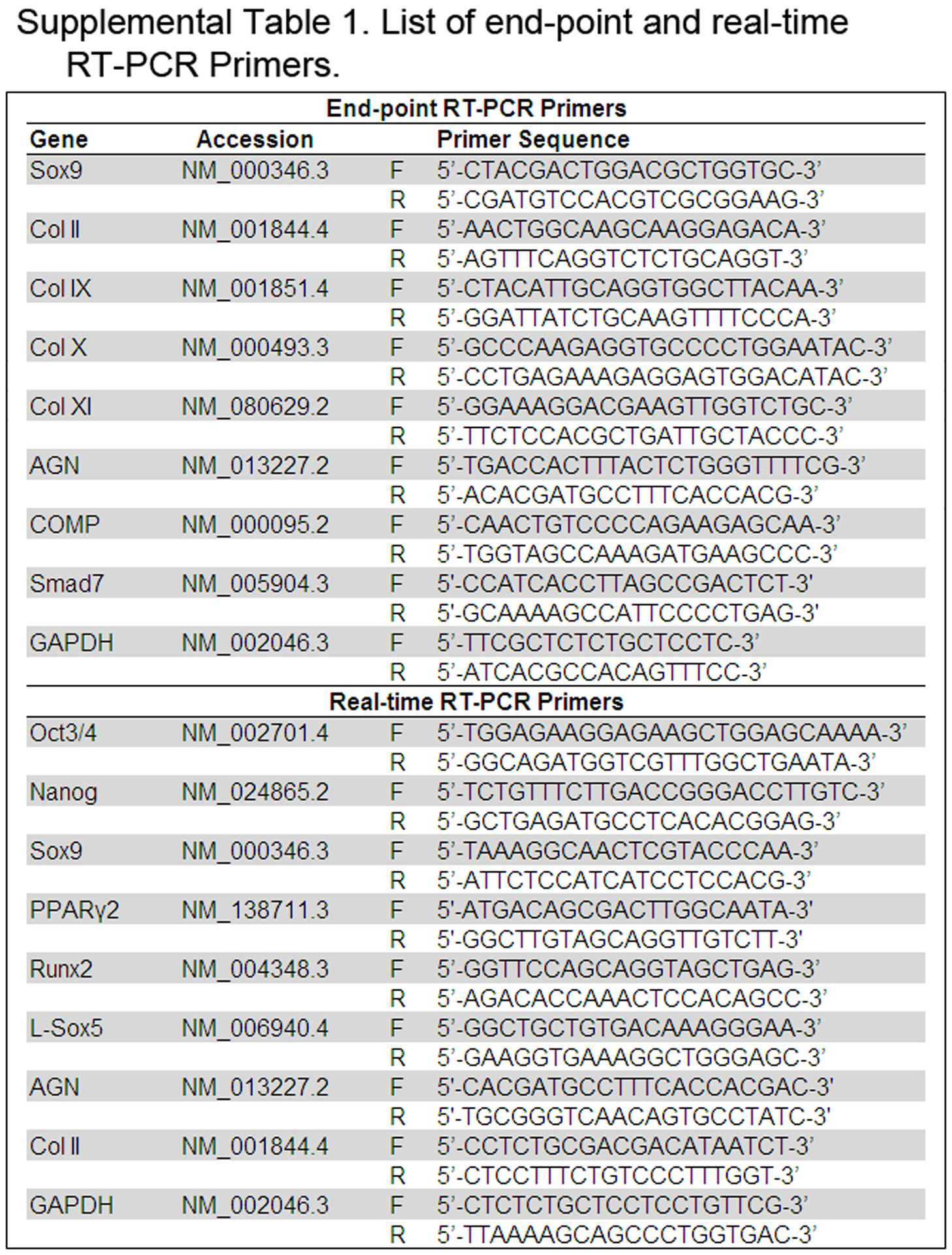

Supplement: Table S1 — List of end-point and real-time RT-PCR primers. (TIF) [file pone.0022887.s004.tif]
